# Impact of COVID-19 on door-to-wire time in ST-segment elevation myocardial infarction treatment: the role of digital communication

**DOI:** 10.1186/s12872-025-04618-7

**Published:** 2025-03-12

**Authors:** Changqing Zhong, Shanjun Mao, Shan Tang, Pengfei Zheng, Jianqiang Peng

**Affiliations:** 1 Department of Cardiovascular Medicine, Hunan Provincial People’s Hospital, Changsha, Hunan China; 2 Department of Cardiovascular Medicine, First Affiliated Hospital of Hunan Normal University, Changsha, Hunan China; 3 Clinical Research Center for Heart Failure in Hunan Province, Changsha, Hunan 410006 China; 4 Department of Statistics, Hunan University, Changsha, Hunan 410006 China

**Keywords:** COVID-19, Chest pain center, ST-segment elevation myocardial infarction, D-to-W, Time prediction

## Abstract

**Introduction:**

ST-segment elevation myocardial infarction (STEMI) is a life-threatening cardiovascular emergency necessitating rapid reperfusion. During the COVID-19 pandemic, healthcare providers faced the challenge of ensuring timely STEMI interventions while managing the risk of viral transmission in hospitals. This study aims to analyze changes in the door-to-wire (D-to-W) time for STEMI treatment across three pandemic phases—early pre-epidemic phase (Group C), initial lockdown phase (Group A), and intermediate normalization phase (Group B). It also examines the impact of digital communication tools, collectively referred to as “InterNet^+^” (e.g., Twitter, WeChat), on treatment processes.

**Methods:**

Based on data of 630 STEMI patients treated in Chest Pain Center in a particular hospital in China from 2019 to 2020, changes in D-to-W time in different groups are measured. Time intervals in STEMI treatment process are also predicted by Bayesian statistics approach. The study investigated the influence of InterNet^+^ utilization before and after the pandemic through a questionnaire-based assessment.

**Results:**

For transfer-non-emergency- treatment, the time from first-electrocardiogram to preliminary-diagnosis in Group-A is significantly longer than that in Groups-B and -C (*p* = 0.004, *p* = 0.004); the time from decision-on-intervention to catheterization-room-activation in Group-A and -B is significantly longer than that in Group-C (*p* = 0.003, *p* < 0.001). For transfer-emergency- treatment, the time from first-medical-contact to arterial-puncture in Group-A and -B is remarkably shorter than that in Group-C (*p* = 0.006). Meanwhile, Bayesian method performs well in forecasting time intervals, so it can provide effective assistance for STEMI treatment. The findings from the questionnaire indicated that physicians perceived a significant association between the optimal management of STEMI and an increased frequency of InterNet^+^ tool usage following the pandemic (*p* = 0.019).

**Conclusions:**

The treatment and management of STEMI patients have been in dilemmas and various time intervals of D-to-W are inevitably prolonged during the COVID-19 pandemic. The implementation of InterNet^ +^ tools proved essential for minimizing delays in D-to-W and FMC-to-W times, offering a valuable strategy for enhancing STEMI care amid ongoing pandemic challenges.

**Clinical trial number:**

Not applicable.

**Supplementary Information:**

The online version contains supplementary material available at 10.1186/s12872-025-04618-7.

## Introduction

According to the *Annual Report on Cardiovascular Health and Diseases in China (2023)* released by the National Center for Cardiovascular Diseases (CVD) in 2024, coronary heart disease and myocardial infarction case numbers have grown rapidly in recent years. The report indicates that the number of patients with CVD is estimated to be 330 million [1]. Particularly, the ST-segment elevation myocardial infarction (STEMI) patients suffer higher pre-hospital and in-hospital mortality and incidence of heart failure, carrying increasingly heavy burden of diseases [2–7]. To treat STEMI rapidly and efficiently, chest pain centers (CPCs) have been built at the national, provincial, and city levels for the purpose of centralized and standardized management of patients. This greatly shortens the time from symptom onset to effective rescue, particularly the revascularization time (taking the door-to-wire (D-to-W) time as the main indicator). For instance, the establishment of a CPC in a prominent hospital in China resulted in a noteworthy decrease of 21.9 min in the D-to-W time [8–11]. With the outbreak of COVID-19 in 2019, processes including the admission, examination, and emergency treatment of STEMI patients have undergone a series of uncertain changes [12–24]. In the context of the ongoing pandemic, STEMI treatment has faced a dilemma, balancing timely reperfusion therapy with strict infection control measures.

Digital communication has become an essential component of telemedicine, significantly improving the efficiency of disease management and treatment processes [25–30]. For example, Studencan et al. (2018) evaluated the benefits of implementing a novel smartphone-based communication system, “STEMI,” which led to a substantial reduction in unwanted secondary STEMI transportations and shortened the overall ischemic time [31]. Additionally, telemedicine facilitates the provision of crucial clinical information that supports cardiologists in making diagnoses and differential diagnoses, thereby reducing the need for in-person consultations [32–35]. However, some telemedicine platforms, particularly those designed for specialized purposes, present high usability barriers, are costly, and are not readily accessible in primary healthcare settings or remote areas of developing countries [36, 37]. Taking the SARS epidemic as a case in point, the limitations of available technology at that time—such as reliance on telephone-based communication—were evident, and were less efficient compared to the more advanced digital communication systems currently available for telemedicine [38–40]. This study thus explores the role of a more widely accessible digital communication approach, i.e., InterNet^+^, specifically the WeChat platform in China—in optimizing emergency response processes for STEMI management during the COVID-19 pandemic [41, 42].

This analysis focuses on STEMI patients admitted to the Chest Pain Center of a specific hospital from 2019 to the end of 2020, with data divided into one year before and after the outbreak of the COVID-19 pandemic. This study analyzes changes in the D-to-W time and influences of relevant factors in different prevention and control periods of the pandemic and one year separately before and after outbreak of the COVID-19 pandemic. Based on this, the key time intervals in the STEMI treatment process were predicted to assist clinicians in diagnosis, treatment, and hospital management. Furthermore, the paper evaluates the impact of InterNet^+^-based tools on enhancing STEMI management and discusses the potential benefits as part of effective coping strategies in clinical practice.

## Methods

### Data sources and sample grouping

All data for this study were collected from the Chairman’s Chest Pain Center Hospital in Hunan Province, China. The study included all patients diagnosed with STEMI who were admitted to the center between October 31, 2016, and December 31, 2020. However, this article primarily investigates the impact on STEMI patients before and after the pandemic. Therefore, the main study sample consisted of patients admitted from January 1, 2019, to December 31, 2020, while the remaining samples were solely utilized for comparison or prediction purposes. It is worth noting that the hospital holds the position of Central unit within the alliance of chest pain centers at all levels in the province. Consequently, the cases handled by the chest pain center include referrals from chest pain centers at all levels within the province. Additionally, hospitals at all levels technically adhere to the guidance provided by the unit, ensuring broad representation. According to the adjustment of the public health emergency response level for the prevention and control of the COVID-19 pandemic in Hunan Province, China, January 20, 2020, and March 24, 2020, were selected as the time points to divide the different periods of prevention and control. In this way, the lockdown period (January 20 to March 24, 2020, Group A), normalized prevention and control period (March 25 to December 31, 2020, Group B), and pre-COVID-19 pandemic period (January 1, 2019 to January 20, 2020, Group C) were divided according to the severity of the pandemic. These groups were adopted to explore changes in the D-to-W time of STEMI patients under different pandemic prevention and control conditions. In the meantime, the pandemic had been surfacing since December 2019 and been sufficient attention in China, which might exert certain influences on local medical visits, and hospitals as the frontline of the pandemic also responded more rapidly than the public. Therefore, the STEMI patients were also divided into Group 2019 and Group 2020 to compare changes in the D-to-W time in one year separately before and after the beginning of the pandemic.

### Definitions of various time intervals in the D-to-W time

The analysis focuses on influences of the COVID-19 pandemic on various time intervals of the D-to-W time in STEMI treatment. Based on whether the patient seeks treatment in the emergency department, the D-to-W time is categorized into emergency and non-emergency groups. Among them, all patients undergoing emergency treatment utilized InterNet^+^, and patients undergoing non-emergency treatment also utilized InterNet^+^ to varying extents. In the case of non-emergency treatment, the D-to-W time is divided into time intervals from door to first medical contact (FMC) (D-to-W_1_), from FMC to first electrocardiogram (ECG) (D-to-W_2_), from first ECG to preliminary diagnosis (D-to-W_3_), from preliminary diagnosis to decision on intervention (D-to-W_4_), from decision on intervention to catheterization room activation (D-to-W_5_), from catheterization room activation to arterial puncture (D-to-W_6_), from arterial puncture to wire (D-to-W_7_), and from wire to end of surgery (D-to-W_8_). In the case of emergency treatment, it is divided into D-to-FMC, the time interval from FMC to arterial puncture (FMC-to-Punc), Punc-to-W, and W-to-End. Other variables in the D-to-W process include the time from taking blood to troponin reporting (Troponin), from the start of treatment to signing informed consent (Informed), and from starting to activation of the catheterization room (Start Cath-Act).

### Screening criteria

(1) Patients conformed to the diagnostic criteria of STEMI; (2) the time from disease onset was less than 24 h; (3) emergency percutaneous coronary intervention (PCI) was performed; and (4) various interval variables of the D-to-W time met processes of emergency treatment groups or non-emergency treatment groups.

### The application of InterNet^+^ in epidemic coping strategies

“InterNet^+^” refers to immediate communication methods employed between teritiary and secondary hospitals, enabling timely and effective telemedicine interactions. This communication mechanism takes advantage of the convenience and interconnectedness of the Internet, facilitating contact between medical experts from teritiary hospitals, healthcare workers in secondary or primary care hospitals, and even high-risk cardiovascular patients. It can be activated when a patient with chest pain needs to seek medical attention.

InterNet^+^ refers to the transmission of critical patient information, such as blood pressure, heart rate, blood oxygen levels, electrocardiogram data, and myocardial enzyme levels, from the primary physician to an advanced hospital information platform with PCI qualifications for in-depth analysis and diagnosis. This facilitates the establishment of a direct pathway, bypassing the emergency department, and enables the preparation of individualized treatment plans. The InterNet^+^ discussed in this paper differs from other specialized telemedicine systems [43, 44]. It only requires a smartphone and the download of a free communication app such as Facebook or WeChat to participate in telemedicine interactions. All services, including text, images, videos, and voice calls, are provided free of charge. It serves as a simplified and cost-free version of a comprehensive telemedicine system. The operation of InterNet^+^ involves healthcare professionals from various levels of hospitals, including emergency doctors from the 120 emergency services, who sign patient privacy confidentiality agreements upon joining the group. Additionally, specialist doctors from the chest pain center of teritiary hospitals are available 24/7 to interact with the communication group. The usage of InterNet^+^ encompasses free communication through text, voice, video, images, and voice calls.

### Statistical analysis

Categorical variables were represented as frequencies (percentages). Continuous variables were expressed as mean ± standard deviation (SD), and the abnormal distribution was represented by the median (interquartile range [IQR]). Chi-squared tests or Fisher’s precise tests were conducted on categorical variables; Student *t*-tests or Wilcoxon rank-sum tests were conducted on continuous variables, and ANOVA or Kruskal-Wallis Test for more than two groups, with *p* < 0.05 indicating statistically significant differences. By conducting these, the research compared changes in the D-to-W time of STEMI patients undergoing emergency treatment or non-emergency treatment and in different modes of admission in different prevention and control periods of the pandemic or before and after the start of the pandemic.

The utilization of big data analysis tools plays a crucial role in effectively optimizing time management strategies implemented in the STEMI process. Based on the Bayesian forecasting model, supposing that every adjacent time intervals of the D-to-W time follow multivariate normal distribution, then the time in the next stage can be predicted according to that in the previous stage [45]. The specific model methodology is elaborated upon in the Supplementary Material S1.

This study employed a questionnaire methodology to gather data on the frequency of InterNet^+^ usage by doctors before and after the epidemic, as well as their perspectives on the effectiveness of STEMI optimization management. The questionnaire design underwent expert review and pre-testing procedures to ensure its precision and dependability. The questionnaire was distributed comprehensively across both teritiary and secondary hospitals, encompassing departments such as cardiovascular medicine, emergency medicine, respiratory medicine, cardiovascular surgery, and other departments directly related to the STEMI process. Participants included attending physicians, treating physicians, residents, and other emergency doctors and/or cardiologists at various levels. Simultaneously, these experts are individuals who possessed a preliminary understanding of InterNet^+^ prior to the pandemic and actively engaged in their respective roles during the pandemic. Subsequently, we performed statistical analysis and interpretation of the collected questionnaire data to examine the efficacy of InterNet^+^ utilization in STEMI optimization management and to provide substantiation for the research objectives.

## Results

### Factors influencing changes in D-to-W

Influencing factors of the D-to-W time were analyzed in the following three scenarios: self-admission–non-emergency treatment, transfer–non-emergency treatment, and transfer-emergency treatment.

**Fig. 1:**
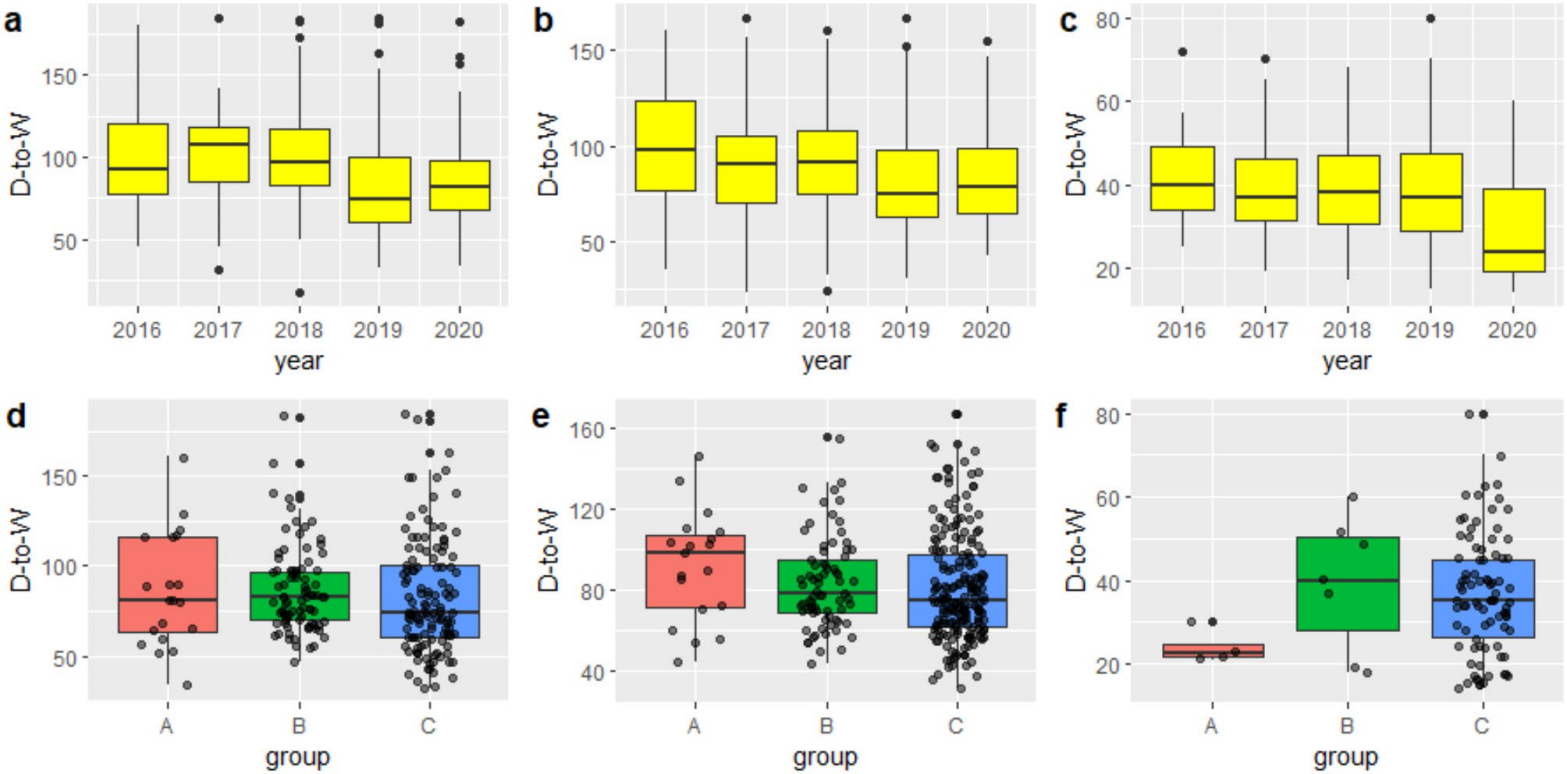
The (**a**,** b**,** c**,** d**,** e**, and **f**) charts represent box plots of D-to-W time of Groups A, B, and C in 2016 to 2020 in the three scenarios of self-admission–non-emergency treatment, transfer–non-emergency treatment, and transfer–emergency treatment, respectively

Figure 1 shows box plots of the D-to-W time of STEMI patients in the five years from 2016 to 2020 and in different prevention and control periods of the pandemic (Groups A, B, and C) during emergency PCI in three different scenarios. Comparison between plots in Fig. 1 reveals that the total D-to-W time decreased from 2016 to 2019 in the scenario of non-emergency treatment (Fig. 1a and **b**), while after outbreak of the pandemic at the end of 2019, the D-to-W time was prolonged in 2020. The D-to-W time in Groups A and B was also longer than that in Group C in different prevention and control periods of the pandemic (Fig. 1d and **e**). In the scenario of emergency treatment (Fig. 1c and **f**), the D-to-W time was reduced in 2020, showing differences with that in the scenario of non-emergency treatment.

A total of 630 cases and the individuals undergoing three different STEMI treatment ways were tested and the results are listed in Table 1. More detailed clinical information and test results under the different treatment ways are presented in Table 1 of Supplemental Material. Comparisons show that different groups as well as Groups 2019 and 2020 exhibit significant differences in the D-to-W time and interval variables. Table 2 displays the one-sided *t*-test results for Groups A-B, Groups A-C, Groups B-C, and Groups 2019–2020. The upper part of Table 2 mainly displays interval variables with significant increases and the lower part mainly shows those with a significant decline.

**Table 1:** Comparison of general clinical information under two subgroups

Variable	All	Subgroup 1			*p* ^a^	Subgroup 2		*p* ^b^
		A	B	C		2019	2020	
Total STEMI cases	630	45	168	417		390	240	
Male	467(74.36%)	27(60%)	129(76.79%)	311(74.58%)	0.06	296(75.9%)	171(71.25%)	0.302
Age	62.08 ± 11.65	59.42 ± 10.75	60.82 ± 12.4	62.89 ± 11.36	0.042	62.96 ± 11.41	60.67 ± 11.91	0.018
D-to-W	73(56-96.75)	87(60–110)	79.5(68–97)	70(51–92)	< 0.001	70(52–94)	77.5(62–98)	0.098
STEMI treatment ways								
Self-admission–Non-emergency	233	21	85	127		118	115	
D-to-W	80(65–102)	81(64–116)	83(70–97)	75(60.5-104.5)	0.408	74.5(61-104.75)	82(68-98.5)	0.742
Transfer–Non-emergency	295	20	76	199		192	103	
D-to-W	77(64–100)	99.5(71.5-108.5)	78.5(68.75–96.5)	75(62.5–98.5)	0.096	75(63–100)	82(65.5-100.5)	0.472
Transfer–Emergency	102	4	7	91		80	22	
D-to-W	36(24.5-48.75)	22.5(21.75–24.75)	40(28-50.5)	37(28–49)	0.13	38.5(29.75–50.25)	24(19.25-39)	0.001

Values are n, n (%), mean ± SD, or median (interquartile range). ^a^The Student t-tests or Wilcoxon rank-sum tests was used. ^b^The ANOVA or Kruskal-Wallis Test was used. ^†^Only STEMI data in Transfer–Emergency treatment was used. D-to-W = the door-to-wire time

**Table 2:** The results of the one-sided test

Variable	Self-admission- Non-emergency				Transfer- Non-emergency				Transfer- Emergency treatment
One-sided test	A-B	A-C	B-C	20 − 19	A-B	A-C	B-C	20 − 19	20 − 19
D-to-W			B > C (0.027)	20 > 19 (0.037)					20 < 19 (0.003)
D-to-W_3_			B > C (0.002)		A > B (0.004)	A > C (0.004)			
D-to-W_5_		A > C (0.003)	B > C (< 0.001)	20 > 19 (< 0.001)		A > C (< 0.001)	B > C (< 0.001)	20 > 19 (< 0.001)	
D-to-W_8_			B > C (0.008)	20 > 19 (0.026)			B > C (0.004)	20 > 19 (0.002)	
Start Cath-Act			B > C (< 0.001)	20 > 19 (< 0.001)			B > C (< 0.001)	20 > 19 (< 0.001)	
D-to-W_1_		A < C (0.004)	B < C (< 0.001)	20 < 19 (< 0.001)		A < C (0.001)	B < C (< 0.001)	20 < 19 (< 0.001)	20 < 19 (< 0.001)
D-to-W_2_				20 < 19 (0.013)			B < C (< 0.001)	20 < 19 (0.011)	
D-to-W_4_	A < B (0.022)	A < C (< 0.001)	B < C (< 0.001)	20 < 19 (< 0.001)	A < B (0.022)	A < C (< 0.001)	B < C (< 0.001)	20 < 19 (< 0.001)	
D-to-W_6_							B < C (< 0.001)	20 < 19 (0.003)	
D-to-W_7_	A < B (0.018)						B < C (0.031)	20 < 19 (0.001)	
FMC-to-Punc									20 < 19 (0.006)
Troponin		A < C (0.033)	A < C (0.007)	20 < 19 (0.003)		A < C (0.045)	A < C (0.024)	20 < 19 (0.020)	
Informed			B < C (< 0.000)				B < C (0.002)	20 < 19 (0.004)	20 < 19 (0.004)
Start Cath-Act	A < B (0.011)				A < B (0.014)				20 < 19 (< 0.001)

Values are the p value of the one-sided test, and only significant results (< 0.05) are shown. D-to-W = the door-to-wire time; D-to-W_1_ = time from door to first medical contact (FMC); D-to-W_2_ = time from FMC to first electrocardiogram (ECG); D-to-W_3_ = time from first ECG to preliminary diagnosis; D-to-W_4_ = time from preliminary diagnosis to decision on intervention; D-to-W_5_ = time from decision on intervention to catheterization room activation; D-to-W_6_ = time from catheterization room activation to arterial puncture; D-to-W_7_ = time from arterial puncture to wire; D-to-W_8_ = time from wire to end of surgery; Troponin = time from taking blood to troponin reporting; Informed = time from the start of treatment to signing informed consent; Start Cath-Act = time from starting to activation of the catheterization room

### Prediction results

**Fig. 2:**
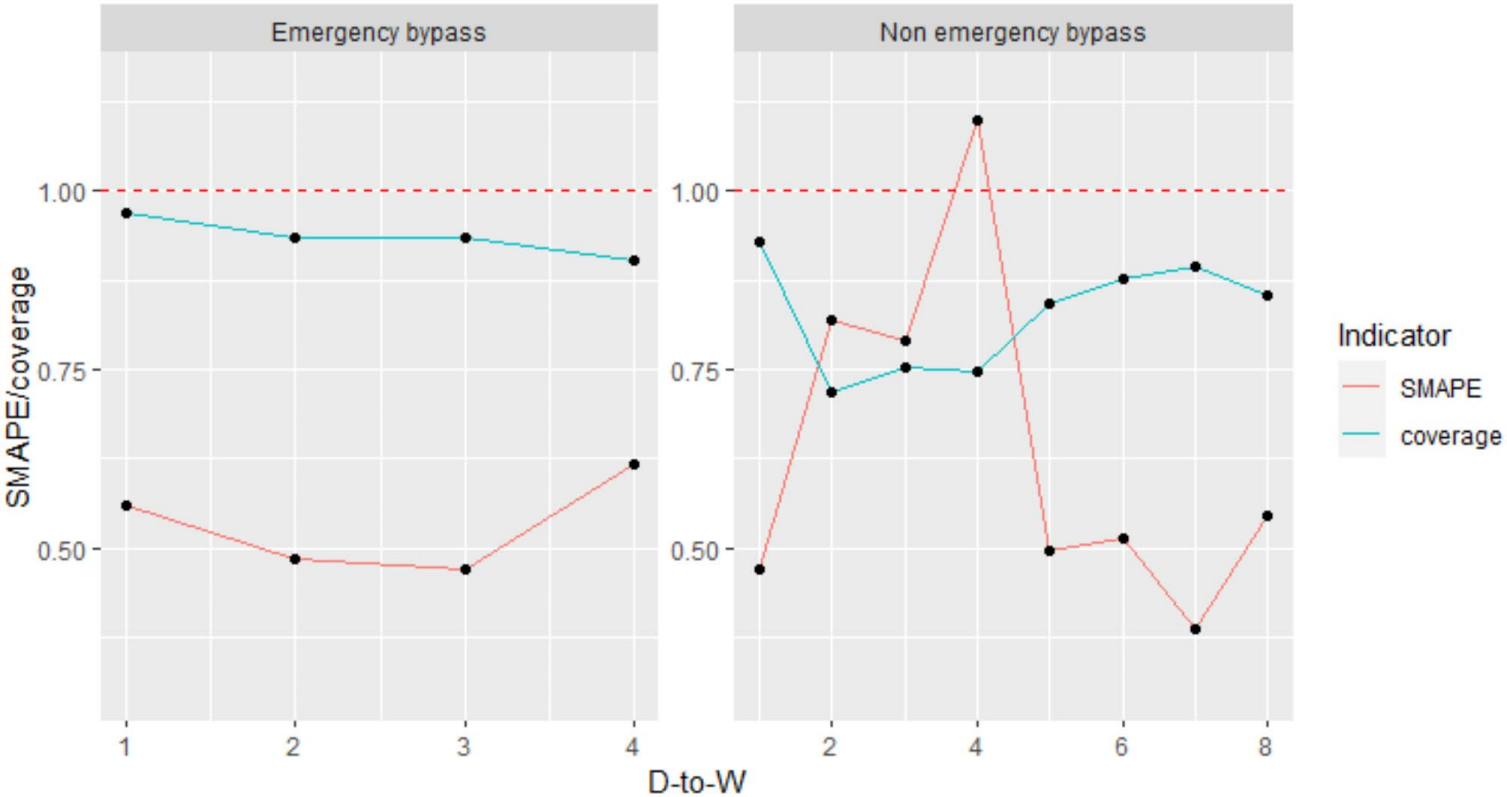
Predictions for emergency treatment and non-emergency treatment scenarios

A total of 1005 STEMI patients undergoing emergency PCI from 2018 to 2020 were selected for prediction, in which 155 with emergency treatment and 850 with non-emergency treatment. The prediction results are shown in Fig. 2, the symmetric mean absolute percentage error (SMAPE) and the confidence interval coverage are adopted as the prediction indicators [46]. The predictive effect is satisfactory if the SMAPE is lower than 100%, and the closer the latter is to 1, the better the prediction. Favorable predictive effects are obtained in the scenario of emergency treatment, with the SMAPE being below 0.6. The predictive effects in the non-emergency treatment scenario are also relatively good, with the coverage being above 0.75. In the scenario of non-emergency treatment, the D-to-W_4_ time is poorly predicted, which is the only variable with the prediction indicator SMAPE greater than 100%.

### Questionnaire analysis

We distributed 70 questionnaires to over 100 relevant physicians in departments associated with chest pain centers at both upper and basic hospitals, and collected 62 responses regarding the utilization of InterNet^+^ (Response rate 88.6%). The demographics from the questionnaire is presented in Table 3. Among them, 53.2% pertained to questionnaires from regional medical centers (teritiary hospitals) and 46.8% were from primary hospitals (secondary hospitals). The proportion of attending physicians and treating physicians was 88.7%.

**Table 3:** Demographic information in the questionnaire

Variables	Total	Regional Medical Center (upper-level hospitals)	Primary Hospitals (basic-level hospitals)	*P*-value^a^
No. Participants	62	33	29	
Gender (F/M)	54.1 / 47.5	69.7 / 30.3	34.5 / 65.5	0.012
Age	31.0 ± 6.1	30.0 ± 6.8	32.0 ± 5.1	0.200
Department (%)	Cardiology Department (52.5%)	Cardiology Department (54.5%)	Cardiology Department (48.3%)	
	General Internal Medicine Department (9.8%)	General Internal Medicine Department (12.1%)	Emergency Medicine Department (13.8%)	
	Others (37.7%)	Others (33.3%)	Others (37.9%)	
Physician Title (%)	Resident Physician (39.3%)	Resident Physician (54.5%)	Attending Physician (65.5%)	
	Attending Physician (36.1%)	Associate Chief Physician (15.2%)	Resident Physician (20.7%)	
	Others (24.6%)	Others (30.3%)	Others (13.8%)	
InterNet^+^ Usage Frequency (times)				
Before Pandemic	16.0 ± 11.6	17.2 ± 11.9	14.7 ± 11.4	0.410
After Pandemic	26.1 ± 13.0	24.6 ± 14.8	27.8 ± 10.7	0.335
Effect (P/N) ^c^	83.9 / 16.1	78.8 / 21.2	89.7 / 10.3	0.312
P-value ^b^	< 0.001	0.028	< 0.001	

^a^P-values for tests conducted in Regional Medical Center and Primary Hospitals

^b^P-values of tests assessing the frequency of InterNet^+^ usage before and after the pandemic

^c^"P” indicates a positive effect, while “N” indicates no positive effect or unclear effect

**Fig. 3:**
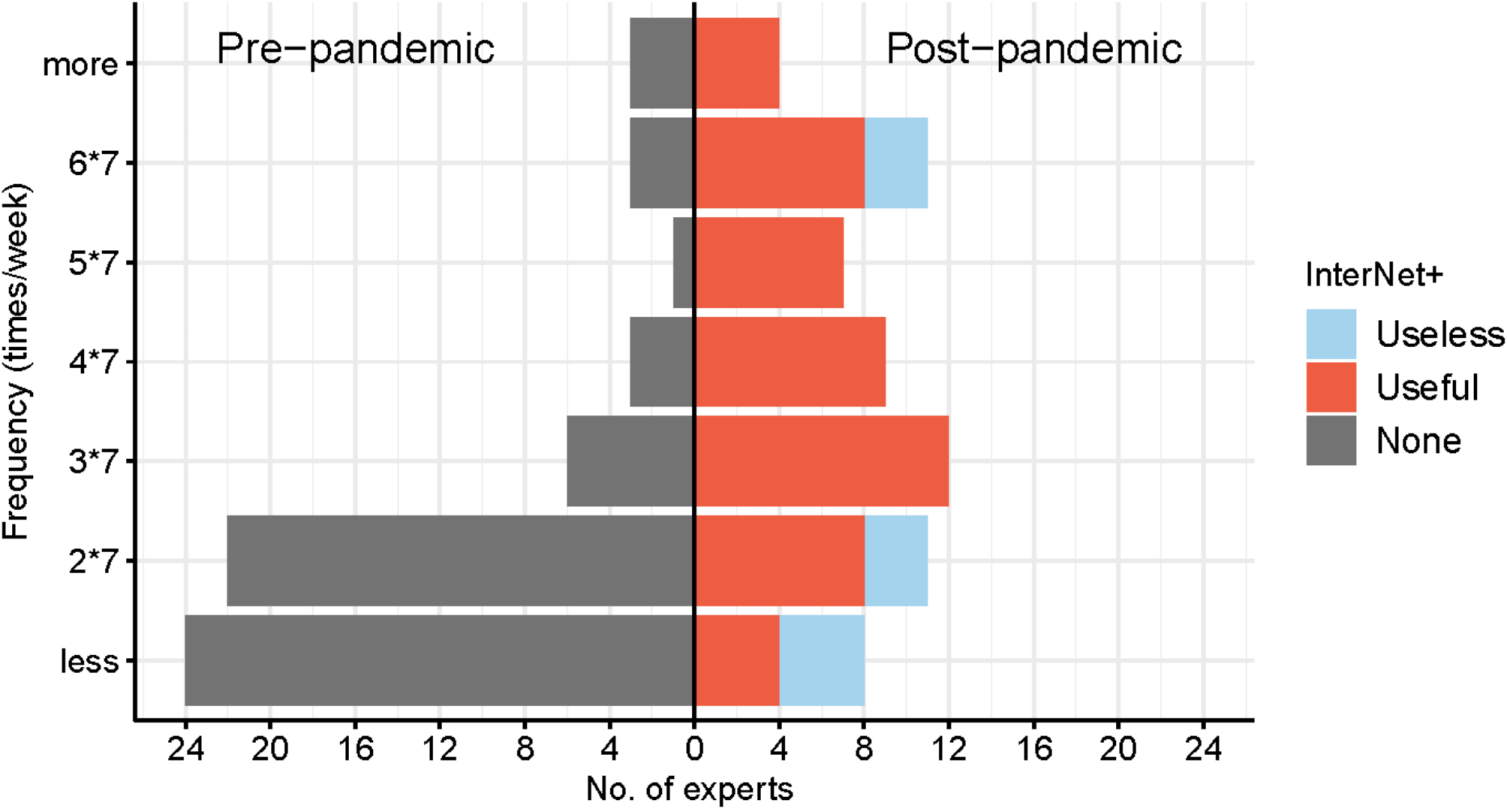
A bar chart illustrating the usage of InterNet^+^ tools by experts and the results of effective judgment on STEMI optimization management is presented. he left and right halves of the chart show the situation before and after the pandemic, respectively. The horizontal axis represents the number of experts, while the vertical axis represents the frequency of InterNet^+^ tool usage per week

Figure 3 illustrates a bar chart representing the frequency of InterNet^+^ usage by doctors before and after the pandemic, as well as their perception of the effectiveness of these tools after the pandemic. It is evident that prior to the epidemic, the utilization of InterNet^+^ was generally low, with most doctors (74.2%) reporting usage of less than 14 (2 × 7) times per week. However, following the epidemic, the majority of doctors (69.4%) exhibited an increase in frequency, with usage exceeding 21 (3 × 7) times per week. Moreover, we discovered that the effectiveness of InterNet^+^ in optimizing STEMI management was significantly associated with the frequency of post-epidemic usage (*p* = 0.019). A more detailed description of the results is illustrated in Supplementary Material S3.

## Discussion

### The analysis of D-to-W time

Amid the ongoing COVID-19 pandemic, the treatment and management of STEMI patients have faced a dilemma because a compromise needs to be made between the timely reperfusion therapy and the rigorous prevention and control of COVID-19 infections [36, 47]. This study found that the use of InterNet^+^ enables doctors to manage STEMI patients more efficiently, with its advantages becoming particularly evident during the pandemic.

1) In the scenario of a transfer–non-emergency treatment, the D-to-W_3_ time among interval variables in Group A is significantly longer than that in Groups B and C (*p* = 0.004, *p* = 0.004). This is probably because, during the initial outbreak of the pandemic, the coping strategies for the pandemic and scientific norms and procedures for sifting COVID-19 infections had not been made yet. Consequently, COVID-19 infected patients could only be identified using a variety of complex methods and indicators, including clinical symptoms, nucleic acid test results, and CT scans of the lungs. This substantially prolonged the time required for diagnosis. After entering the normalized prevention and control period, standardized procedures and contingency plans have been implemented. Especially, the application of the InterNet^+^ and big data analysis to the pandemic have significantly reduced the time needed for COVID-19 prevention and control.

2) In the scenario of non-emergency treatment, the interval variable D-to-W_5_ time in Groups A and B is obviously longer than that in Group C (*p* = 0.003, *p* < 0.001). The procedures and regulations related to pandemic prevention and control have been implemented to the stage compared with the pre-pandemic period. For example, epidemiological survey and nucleic acid test results need to be added in the stage. Emergency procedures and disinfection for pandemic prevention and control are introduced in the catheterization room [48, 49]. During the pandemic outbreak and subsequent prevention and control period, additional pandemic prevention and control measures are implemented, resulting in increased D-to-W time intervals. Therefore, new methods and procedures need to be developed in a bid to reduce the delay in D-to-W amid the pandemic.

3) In the scenario of emergency treatment, results show that the interval variable FMC-to-Punc time in the Group 2020 during the COVID-19 pandemic is markedly shorter than the pre-pandemic Group 2019 (*p* = 0.006). Due to prevention and control measures during the COVID-19 pandemic, many STEMI patients choose to have a telemedicine-type interaction over the InterNet^+^ in the diagnosis process. For example, the first-contact doctors in basic-level hospitals send clinical information and key examination and inspection results of patients to the InterNet^+^ before, and during the transfer process to interact with cardiovascular specialists in upper-level hospitals. In this way, the involvement of specialists from upper-level hospitals in the transfer process significantly reduces the FMC-to-Punc time during emergency treatment, thereby improving treatment efficiency. Emergency treatmentdata show that even in the prevention and control period of the pandemic, the D-to-W time can also be reduced as long as more patients use the telemedicine interaction function of the InterNet^+^. This results in effects similar to those observed in other studies at present [50–52].

4) Favorable prediction results are attained for the scenario of emergency treatment, with the SMAPE basically below 0.6; the prediction results for the scenario of non-emergency treatment are also relatively good, with the coverage exceeding 0.75. In the scenario of a non-emergency treatment, the D-to-W_4_ time is poorly predicted, which is the only variable with the prediction indicator SMAPE exceeding 100%. This is probably because the time for deciding whether or not to intervene in cases involving Chinese patients is closely related to the judgement and decision of family members. However, family members exhibit greater individual differences than patients, which is related to many complex factors such as the family composition, level of education and trust in hospitals and doctors, so the prediction results are poor [53–55]. The InterNet^+^ has been applied to all patients undergoing emergency treatment, so the condition information of STEMI patients is presented as pictures or texts to specialist physicians in teritiary hospitals in advance. As a result, the diagnosis of patients is transmitted in a timely manner, allowing them to proceed to the next stage of treatment promptly and ensuring timely medical intervention. In addition, because the preoperative conversation with family members also moves forward to basic-level hospitals and ambulances, the stage with the longest delay (D-to-W_4_) is also significantly shortened in the case of an emergency treatment (*p* = 0.004).

### The beneficial impact of InterNet^+^ in coping strategies

The core to treatment of STEMI is to minimize the time from disease onset to effective revascularization. The standardized operation of each chest pain center before the COVID-19 pandemic has allowed a decrease in the mortality of STEMI patients. Despite this, the out-of-hospital mortality is significantly higher than in-hospital mortality of STEMI patients [56]. In particular, the COVID-19 pandemic unavoidably prolongs the rescue time including the D-to-W time of STEMI patients. In such context, strengthening out-of-hospital links of chest pain centers, paying attention to seamless pre-admission and in-hospital connection, and advancing the whole-process management system have become pressing issues. For instance, a meta-analysis investigating the tangible impacts of pre-hospital triage utilizing telemedicine for STEMI patients revealed that pre-hospital triage via telemedicine was associated with a nearly 50% reduction in the time spent on STEMI treatment [37].

The D-to-W time is shortened in the scenario of emergency treatment while it is prolonged significantly in scenarios involving non-emergency treatment after the pandemic: this is mainly ascribed to the contribution of telemedicine interaction of the InterNet^+^. The research results indicate that the InterNet^+^ is a key to overcoming this bottleneck. Similarly, cases utilizing telemedicine (such as The Latin America Telemedicine Network (LATIN) and China’s Tiantanzhixin application) in diverse regions demonstrate that comparable InterNet^+^ tools can effectively diminish D-to-W time and enhance the efficacy of patient care [50, 57]. Based on our questionnaire survey conducted among doctors at various levels of hospitals, it was found that there was an increased utilization of InterNet^+^ during the pandemic. Additionally, it was observed that patients with myocardial infarction received faster and more effective PCI treatment in hospitals, attributed to the shortened door-to-wire (D-to-W) time achieved through the application of InterNet^+^ tools (*p* = 0.019). This finding agrees with results of other observational studies and meta-analysis, which show that telemedicine can shorten the pre-hospital delays of patients receiving direct PCI [58]. The telemedicine also provides additional clinical information, which can assist cardiologists in diagnosis and differential diagnosis with no need for an in-person diagnostic interview [32]. However, the InterNet^+^ mentioned in the research is different from those specially developed telemedicine systems. In comparison, other specially developed telemedicine software has a high threshold to use and is expensive, making it inaccessible to primary hospitals and remote regions in developing countries. The InterNet^+^ used in this study has unique attributes, such as accessibility, simplicity, real-time communication, and cost-effectiveness, which distinguish it from more traditional or advanced remote platforms.

In summary, the InterNet^+^ as a simplified and free-charge telemedicine system, has the following two main functions: (1) It is key to reducing the effective rescue time of STEMI patients and therefore is worthy of further popularization, standardization, and improvement; (2) It also serves an effective tool for overcoming the bottleneck of time delay in STEMI rescue under the current COVID-19 pandemic prevention and control measures. Use of the InterNet^+^ by more patients should be advocated against the background of current normalized prevention and control measures for the pandemic. If the high-risk group of myocardial infarction can be managed by incorporating them in the telemedicine of the InterNet^+^ in the future, the effective control of the rescue time for myocardial infarction can be brought forward to the time from disease onset to FMC. This will open the last mile of the “expressway” for rescuing myocardial infarction patients and revolutionarily improving the prognosis of myocardial infarction. A more comprehensive discussion is provided in Supplementary Material S3.

### Limitations

The methods and analyses presented in this paper did have several limitations which could be improved in the future: (1) Groups A, B and C can be divided more convincingly, for example, after the COVID-19 pandemic, the completed data can be modeled, and the period of the outbreak can be segmented in a more objective way; (2) The discussion of the use of the InterNet^+^ can be more detailed, such as how to ensure the privacy of patients, can build a platform for both of doctors and patients; (3) The patients included in this study were solely from a chest pain hospital in China. It is worth noting that chest pain centers in other countries may have implemented different protocols during the epidemic, which could have had varying effects on different metrics related to STEMI treatment.

## Conclusions

Due to the COVID-19 pandemic, the treatment and management of STEMI patients have been in a dilemma and various time intervals of the D-to-W time are inevitably prolonged. However, the application of InterNet^+^ not only shortens the D-to-W time in hospitals in STMEI management but also will be key to reducing the FMC-to-W time outside hospitals, regardless of the impact of the COVID-19 pandemic.

## Electronic supplementary material

Below is the link to the electronic supplementary material.

Supplementary Material 1

## Data Availability

The data underlying this article were provided by a particular hospital of China, under licence / by permission. Data will be shared on request to the corresponding author with permission of the particular hospital.

## Abbreviations

COVID-19: Coronavirus disease-2019
D-to-W: Time from the door of hospital to wire crossing
FMC-to-W: Time from first medical contact to wire crossing
PCI: Percutaneous coronary intervention
SMAPE: Symmetric mean absolute percentage error
