# Supplementary material for: Impact of COVID-19 on door-to-wire time in ST-segment elevation myocardial infarction treatment: the role of digital communication
Source: BMC Cardiovasc Disord. 2025 Mar 12;25:173. doi: 10.1186/s12872-025-04618-7 (PMC11899889; doi:10.1186/s12872-025-04618-7)
Supplement: Supplementary file 1 — Supplementary Material 1 [file 12872_2025_4618_MOESM1_ESM.docx]

Supplement to “**Impact of COVID-19 on Door-to-Wire Time in STEMI Treatment: The Role of Digital Communication**”

Contents

[**S1. The Bayesian forecasting model** 1](#_Toc161563005)

[**S2. More details of the Results** 3](#_Toc161563006)

[**S2.1 Factors influencing changes in D-to-W** 3](#_Toc161563007)

[**S2.2 Prediction results** 8](#_Toc161563008)

[**S3. More details of the Discussion** 8](#_Toc161563009)

[**The analysis of D-to-W time** 8](#_Toc161563010)

[**The Beneficial impact of InterNet+ in coping strategies** 11](#_Toc161563011)

[**S4 Questionnaire questions and options** 15](#_Toc161563012)

[**References** 17](#_Toc161563013)

**S1. The Bayesian forecasting model**

It is assumed that any two adjacent time intervals of the D-to-W time follow a multivariate normal distribution, that is, $y\sim N_{p}(\theta,\Sigma), p=2$, in which $\text{y}=\left(\begin{matrix} y_{1} \\ y_{2} \end{matrix}\right), \text{θ}=\left(\begin{matrix} \theta_{1} \\ \theta_{2} \end{matrix}\right), \Sigma\text{=} \left(\begin{matrix} \sigma_{1}^{2} & \sigma_{2,1} \\ \sigma_{1,2} & \sigma_{2}^{2} \end{matrix}\right)$. The independent and identical distribution of *n* samples obeys a multivariate normal distribution, so the joint probability density function is

$$\begin{matrix} p(\text{y}_{1},\cdots,\text{y}_{n}\left|\text{θ},\right.\Sigma)=\prod_{i=1}^{n} (2\pi)^{-p/2}|\Sigma|^{-1/2}exp\{-(\text{y}_{i}-\text{θ})^{T}\Sigma^{-1}(\text{y}_{i}-\text{θ})/2\} \\ =(2\pi)^{-np/2}|\Sigma|^{-n/2}exp\{-\frac{1}{2}\sum_{i=1}^{n} (\text{y}_{i}-\text{θ})^{T}\Sigma^{-1}(\text{y}_{i}-\text{θ})\} \\ \propto\exp\{-\frac{1}{2}\text{θ}^{T}n\Sigma^{-1}\text{θ}+\text{θ}^{T}n\Sigma^{-1}\bar{\text{y}}\}, \end{matrix}$$

where . Meanwhile, given the priors of $\text{θ},\Sigma$ as follows

$$\begin{matrix} \text{θ}\sim N_{p}(\text{μ}_{0},\Lambda_{0}), \\ \Sigma\sim W_{p}^{-1}(v_{0},\text{S}_{0}^{-1}), \end{matrix}$$

then the posterior distribution of parameters is expressed as

$$\begin{matrix} p(\text{θ}\left|\text{y}_{1},\cdots,\text{y}_{n},\right.\Sigma)\propto\exp\{-\frac{1}{2}\text{θ}^{T}\Lambda_{0}^{-1}\text{θ}+\text{θ}^{T}\Lambda_{0}^{-1}\text{μ}_{0}\}\times\exp\{-\frac{1}{2}\text{θ}^{T}n\Sigma^{-1}\text{θ}+\text{θ}^{T}n\Sigma^{-1}\bar{\text{y}}\} \\ =\exp\{-\frac{1}{2}\text{θ}^{T}(\Lambda_{0}^{-1}+n\Sigma^{-1})\text{θ}+\text{θ}^{T}(\Lambda_{0}^{-1}\text{μ}_{0}+n\Sigma^{-1}\bar{\text{y}})\}, \\ p(\Sigma\left|\text{y}_{1},\cdots,\text{y}_{n},\right.\text{θ})\propto p(\Sigma)\times p(\text{y}_{1},\cdots,\text{y}_{n}\left|\text{θ},\right.\Sigma) \\ \propto(|\Sigma|^{-(v_{0}+p+1)/2}\exp\{-tr(\text{S}_{0}\Sigma^{-1})/2\})\times(|\Sigma|^{-n/2}\exp\{-tr(\text{S}_{\theta}\Sigma^{-1})/2\}) \\ = |\Sigma|^{-(v_{0}+n+p+1)/2}\exp\{-tr([\text{S}_{0}+\text{S}_{\theta}]\Sigma^{-1})/2\}, \end{matrix}$$

where $\text{S}_{\theta}=\sum_{i=1}^{n} (\text{y}_{i}-\text{θ})(\text{y}_{i}-\text{θ})^{T}$.

According to Formula (3), the two conditional posteriori distributions can be written as

$$\begin{matrix} \text{\{θ}\left|\text{y}_{1},\cdots,\text{y}_{n},\right.\Sigma\}\sim N_{p}(\text{μ}_{n},\Lambda_{n}), \\ \text{\{}\Sigma\left|\text{y}_{1},\cdots,\text{y}_{n},\right.\text{θ}\}\sim W_{p}^{-1}(v_{n},\text{S}_{n}^{-1}), \end{matrix}$$

where $\text{μ}_{n}=(\Lambda_{0}^{-1}+n\Sigma^{-1})^{-1}(\Lambda_{0}^{-1}\text{μ}_{0}+n\Sigma^{-1}\bar{\text{y}}), \Lambda_{n}=(\Lambda_{0}^{-1}+n\Sigma^{-1})^{-1}, v_{n}=v_{0}+n, \text{S}_{n}^{-1}=[\text{S}_{0}+\text{S}_{\theta}]^{-1}$.

The Monte Carlo method is then adopted to estimate the parameters. The Gibbs sampling process is shown as follows:

1. $\text{θ}^{(s+1)}$ is sampled from the conditional posterior distribution of $\text{θ}$: $\text{μ}_{n}$ and $\Lambda_{n}$ are calculated according to $\text{y}_{1},\cdots,\text{y}_{n}$ and $\Sigma^{(s)}$; $\text{θ}^{(s+1)}\sim N_{p}(\text{μ}_{n},\Lambda_{n})$ is sampled.

2. $\Sigma^{(s+1)}$ is sampled from the conditional posterior distribution of $\Sigma$: $\text{S}_{n}$ is calculated according to $\text{y}_{1},\cdots,\text{y}_{n}$ and $\text{θ}^{(s+1)}$; $\Sigma^{(s+1)}\sim W_{p}^{-1}(v_{0}+n,\text{S}_{n}^{-1})$ is sampled.

Given initial parameter values of the prior distribution, this sampling process is repeated to screen samples that meet conditions. In this way, the sampling mode is obtained for prediction. The symmetric mean absolute percentage error (SMAPE) and the confidence interval coverage are adopted as the prediction indicators ^[1]^. The predictive effect is satisfactory if the SMAPE is lower than 100%, and the closer the latter is to 1, the better the prediction, where $SMAPE \text{=} \frac{100\text{\%}}{n}\sum_{i=1}^{n} \frac{\left|\hat{y}_{i}-y_{i}\right|}{(\left|\hat{y}_{i}\right|+\left|y_{i}\right|)/2}$.

For individuals to be predicted, the time in the (*i+*1)^th^ stage needs to be predicted given the time in the *i*^th^ stage of D-to-W. At first, 30 latest individuals are selected from the database (training set) and trained, thus obtaining the sampling model. Then, the predicted time in the (*i+*1)^th^ stage and the confidence interval are attained using the trained sampling model according to the time in the *i*^th^ stage of the individuals.

**S2. More details of the Results**

**S2.1 Factors influencing changes in D-to-W**

A total of 630 cases and the individuals undergoing three different STEMI treatment ways were tested and the results are listed in Table S.1. Table S.2 displays the one-sided *t*-test results for Groups A-B, Groups A-C, Groups B-C, and Groups 2019-2020. The upper part of Table S.2 mainly displays interval variables with significant increases and the lower part mainly shows those with a significant decline.

Table S.1. Comparison of general clinical information under two subgroups

| Group | Variable | All | A | B | C | *p* | 2019 | 2020 | *p* |
| --- | --- | --- | --- | --- | --- | --- | --- | --- | --- |
| Total cases | *N* | 630 | 45 | 168 | 417 |  | 390 | 240 |  |
|  | Male | 467(74.36%) | 27(60%) | 129(76.79%) | 311(74.58%) | 0.06 | 296(75.9%) | 171(71.25%) | 0.302 |
|  | Age | 62.08 ± 11.65 | 59.42 ± 10.75 | 60.82 ± 12.4 | 62.89 ± 11.36 | 0.042 | 62.96 ± 11.41 | 60.67 ± 11.91 | 0.018 |
|  | D-to-W | 73(56-96.75) | 87(60-110) | 79.5(68-97) | 70(51-92) | < 0.001 | 70(52-94) | 77.5(62-98) | 0.098 |
|  | D-to-W_1_ | 1(1-2) | 1(1-1) | 1(1-1) | 2(1-2) | < 0.001 | 2(1-2) | 1(1-1) | < 0.001 |
|  | D-to-W_2_ | 2(1-3) | 2(1-3) | 2(1-3) | 2(1-3) | 0.042 | 2(1-3) | 2(1-3) | 0.001 |
|  | D-to-W_3_ | 15(8.75-25) | 17(11-31.75) | 15(8-25) | 15(9-24) | 0.508 | 15(9-24) | 15.5(8-27.25) | 0.462 |
|  | D-to-W_4_ | 4(0-12) | 0(0-1) | 1(0-3) | 8(2-16) | < 0.001 | 9(3-19) | 0(0-2) | < 0.001 |
|  | D-to-W_5_ | 18(10-29) | 25(14-46) | 32(20-41) | 13(7.75-20) | < 0.001 | 13(8-20) | 29(17-40) | < 0.001 |
|  | D-to-W_6_ | 10(7-15) | 10(8-15) | 10(6-14.25) | 10(7-15) | 0.028 | 11(7-15) | 10(6-14.25) | 0.003 |
|  | D-to-W_7_ | 53(36-71) | 13(9-18) | 17(12-22) | 16.5(12-24) | < 0.001 | 17(12-24) | 16(10-21) | < 0.001 |
|  | D-to-W_8_ | 16(11-22) | 27.5(22.75-44) | 31(21.75-46) | 26(17.25-40) | 0.033 | 25(17-40) | 30(21.25-46) | 0.019 |
|  | FMC-to-Punc | 27(19-42) | 64.5(43.75-88.25) | 61(49-75) | 48(31-68) | 0.002 | 49(32-68.25) | 60(45-77) | < 0.001 |
|  | Troponin | 19(19-20) | 19(19-19) | 19(19-20) | 19(19-20) | 0.001 | 19(19-20) | 19(19-20) | 0.001 |
|  | Informed | 7.5(3-15) | 7(4-10) | 6(3-12) | 8(4-15) | 0.08 | 8(4-15) | 6(3-12) | 0.004 |
|  | Start Cath-Act | 13(7-20) | 13.5(5-29.25) | 25(15.75-32) | 10(5-17) | < 0.001 | 10(6-17) | 19(10-30) | < 0.001 |
| Self-admission–  Non-emergency bypass | *N* | 233 | 21 | 85 | 127 |  | 118 | 115 |  |
|  | Male | 176(75.86%) | 12(57.14%) | 63(74.12%) | 101(79.53%) | 0.083 | 96(81.36%) | 80(69.57%) | 0.066 |
|  | Age | 61.64 ± 12.21 | 61.48 ± 10.6 | 61.48 ± 12.82 | 61.78 ± 12.13 | 0.983 | 61.99 ± 12.06 | 61.29 ± 12.4 | 0.661 |
|  | D-to-W | 80(65-102) | 81(64-116) | 83(70-97) | 75(60.5-104.5) | 0.408 | 74.5(61-104.75) | 82(68-98.5) | 0.742 |
|  | D-to-W_1_ | 1(1-2) | 1(1-2) | 1(1-1) | 2(1-2) | < 0.001 | 2(1-2) | 1(1-1) | < 0.001 |
|  | D-to-W_2_ | 2(1-3) | 2(1-3) | 2(1-2) | 2(1-3) | 0.314 | 2(1-3) | 2(1-2) | 0.023 |
|  | D-to-W_3_ | 14(7-22.75) | 13(5-19) | 14(7.5-25.5) | 14(8-21.75) | 0.554 | 14(7-20) | 13(7-26) | 0.729 |
|  | D-to-W_4_ | 2(0-12) | 0(0-2.25) | 1(0-2.5) | 7.5(1-20) | < 0.001 | 10(2-22) | 0.5(0-2) | < 0.001 |
|  | D-to-W_5_ | 22(13-33) | 33(16-46) | 32(21-41) | 16(8.5-23) | < 0.001 | 16(8-23) | 31(19-40.5) | < 0.001 |
|  | D-to-W_6_ | 10(7-15) | 10(8-15) | 10(7-15) | 10(7-16) | 0.452 | 10.5(7-16) | 10(7-15) | 0.29 |
|  | D-to-W_7_ | 60(45-75) | 11(9-16) | 17(13-22) | 15(10-21) | 0.087 | 15(10-20.75) | 16(10.5-21.5) | 0.03 |
|  | D-to-W_8_ | 15(10-21) | 26(21-50) | 30(21-46) | 25(18-40) | 0.037 | 25(18.5-40) | 29(20.5-46) | 0.557 |
|  | FMC-to-Punc | 27(20-44) | 61(45.5-88.5) | 63(53-75) | 58(40-75) | 0.13 | 57.5(40-73.25) | 63(49-79) | 0.045 |
|  | Troponin | 19(19-20) | 19(19-19) | 19(19-20) | 20(19-20) | 0.022 | 20(19-20) | 19(19-20) | 0.017 |
|  | Informed | 8(3-15) | 8(4-16) | 7(3-12) | 10(3-18) | 0.368 | 9.5(3-18.25) | 7(3.5-13) | 0.289 |
|  | Start Cath-Act | 15(8-25) | 13(5-29) | 24(15-32) | 10(6-19.5) | < 0.001 | 11.5(7-20) | 19(10-30) | < 0.001 |
| Transfer–  Non-emergency bypass | *N* | 295 | 20 | 76 | 199 |  | 192 | 103 |  |
|  | Male | 213(72.45%) | 11(55%) | 59(77.63%) | 143(71.86%) | 0.13 | 140(72.92%) | 73(70.87%) | 0.913 |
|  | Age | 62.17 ± 11.25 | 59.9 ± 9.79 | 60.78 ± 11.72 | 62.93 ± 11.18 | 0.237 | 63 ± 11.27 | 60.63 ± 11.11 | 0.084 |
|  | D-to-W | 77(64-100) | 99.5(71.5-108.5) | 78.5(68.75-96.5) | 75(62.5-98.5) | 0.096 | 75(63-100) | 82(65.5-100.5) | 0.472 |
|  | D-to-W_1_ | 1(1-2) | 1(1-1) | 1(1-1) | 1.5(1-2) | < 0.001 | 2(1-2) | 1(1-1) | < 0.001 |
|  | D-to-W_2_ | 2(1-3) | 2(1-3) | 2(1-3) | 2(1-3.25) | 0.191 | 2(1-3) | 2(1-3) | 0.032 |
|  | D-to-W_3_ | 14(9-22.5) | 25.5(14.25-38) | 15(8-25) | 14(8-20.75) | 0.001 | 14(8.75-21.25) | 16(10-26.5) | 0.053 |
|  | D-to-W_4_ | 5(0-13) | 0(0-0) | 1(0-3) | 10(3-18) | < 0.001 | 10(3-19) | 0(0-2) | < 0.001 |
|  | D-to-W_5_ | 17(10-29) | 25(20-47.5) | 33(19.5-41) | 13(10-20) | < 0.001 | 13(9-20) | 31(19-41) | < 0.001 |
|  | D-to-W_6_ | 10(7-15) | 11.5(7.75-18.5) | 9(6-12) | 11(8-15) | 0.006 | 11(8-15) | 10(6-13) | 0.004 |
|  | D-to-W_7_ | 58(44-75) | 16.5(10.75-20.25) | 15(11.5-20) | 18(13-25) | 0.001 | 19(13-25) | 15(11-20) | 0.01 |
|  | D-to-W_8_ | 17(12-24) | 27(22.5-37) | 32(22-47) | 26(16-40) | 0.034 | 25.5(15-40) | 31(22.25-45.5) | 0.005 |
|  | FMC-to-Punc | 28(18.75-41) | 82(53.5-90.5) | 61(52-76) | 53(43-71) | 0.025 | 53(43-73) | 61(49-81) | 0.003 |
|  | Troponin | 19(19-20) | 19(19-19) | 19(19-20) | 19(19-20) | 0.036 | 19(19-20) | 19(19-20) | 0.024 |
|  | Informed | 8(4-16) | 7(5-9.5) | 6(3-12) | 10(5-18) | 0.021 | 10(5-18) | 7(4-11) | 0.006 |
|  | Start Cath-Act | 14(8-20) | 17(9.75-30) | 25.5(17.75-33.25) | 10(6-17) | < 0.001 | 10(6-17) | 23(15.25-32) | < 0.001 |
| Transfer–  Emergency bypass | *N* | 102 | 4 | 7 | 91 |  | 80 | 22 |  |
|  | Male | 78(76.47%) | 4(100%) | 7(100%) | 67(73.63%) | 0.192 | 60(75%) | 18(81.82%) | 0.583 |
|  | Age | 62.85 ± 11.52 | 46.25 ± 8.73 | 53.14 ± 13.66 | 64.33 ± 10.59 | < 0.001 | 64.29 ± 10.74 | 57.64 ± 12.96 | 0.035 |
|  | D-to-W | 36(24.5-48.75) | 22.5(21.75-24.75) | 40(28-50.5) | 37(28-49) | 0.13 | 38.5(29.75-50.25) | 24(19.25-39) | 0.001 |
|  | D-to-FMC | 1(1-2) | 1(0.75-1) | 0(0-1) | 2(1-2) | — | 2(1-2.25) | 0.5(0-1) | < 0.001 |
|  | FMC-to-Punc | 17(10-24) | 10.5(9.75-11.75) | 20(14.5-25.5) | 17(10-24.75) | — | 18(12-26.5) | 10.5(9-19) | 0.253 |
|  | Punc-to-W | 17(11-23) | 12(9.25-15) | 17(10-25.5) | 17(11-24.5) | — | 17(11-25) | 14(10-18) | 0.012 |
|  | W-to-End | 27(18-40) | 36(27.5-60.25) | 27(20.5-42) | 26(18-38) | — | 26(18-38) | 30(26-44) | 0.186 |
|  | Troponin | 19(19-20) | — | 18(18-18) | 19.5(19-20) | — | 19.5(19-20) | 18(18-18) | — |
|  | Informed | 5(2-10.5) | 2(1.5-2.5) | 3(2-11) | 5.5(2-11) | — | 6(4-11) | 2.5(2-4) | 0.008 |
|  | Start Cath-Act | 10(4-15) | 0(0-2.5) | 5(4-15.5) | 10.5(5-15) | — | 12(5-15.25) | 4(0.75-6.75) | 0.001 |

Table S.2. The results of the one-sided test.

| Variable | Self-admission-  Non-emergency bypass | | | | Transfer-  Non-emergency bypass | | | | Transfer-  Emergency  bypass |
| --- | --- | --- | --- | --- | --- | --- | --- | --- | --- |
| One-sided test | A-B | A-C | B-C | 20-19 | A-B | A-C | B-C | 20-19 | 20-19 |
| D-to-W |  |  | B > C  (0.027) | 20 > 19  (0.037) |  |  |  |  | 20 < 19  (0.003) |
| D-to-W_3_ |  |  | B > C  (0.002) |  | A > B  (0.004) | A > C  (0.004) |  |  |  |
| D-to-W_5_ |  | A > C  (0.003) | B > C  (< 0.001) | 20 > 19  (< 0.001) |  | A > C  (< 0.001) | B > C  (< 0.001) | 20 > 19  (< 0.001) |  |
| D-to-W_8_ |  |  | B > C  (0.008) | 20 > 19  (0.026) |  |  | B > C  (0.004) | 20 > 19  (0.002) |  |
| Start Cath-Act |  |  | B > C  (< 0.001) | 20 > 19  (< 0.001) |  |  | B > C  (< 0.001) | 20 > 19  (< 0.001) |  |
|  |  |  |  |  |  |  |  |  |  |
| D-to-W_1_ |  | A < C  (0.004) | B < C  (< 0.001) | 20 < 19  (< 0.001) |  | A < C  (0.001) | B < C  (< 0.001) | 20 < 19  (< 0.001) | 20 < 19  (< 0.001) |
| D-to-W_2_ |  |  |  | 20 < 19  (0.013) |  |  | B < C  (< 0.001) | 20 < 19  (0.011) |  |
| D-to-W_4_ | A < B  (0.022) | A < C  (< 0.001) | B < C  (< 0.001) | 20 < 19  (< 0.001) | A < B  (0.022) | A < C  (< 0.001) | B < C  (< 0.001) | 20 < 19  (< 0.001) |  |
| D-to-W_6_ |  |  |  |  |  |  | B < C  (< 0.001) | 20 < 19  (0.003) |  |
| D-to-W_7_ | A < B  (0.018) |  |  |  |  |  | B < C  (0.031) | 20 < 19  (0.001) |  |
| FMC-to-Punc |  |  |  |  |  |  |  |  | 20<19  (0.006) |
| Troponin |  | A < C  (0.033) | A < C  (0.007) | 20 < 19  (0.003) |  | A < C  (0.045) | A < C  (0.024) | 20 < 19  (0.020) |  |
| Informed |  |  | B < C  (< 0.000) |  |  |  | B < C  (0.002) | 20 < 19  (0.004) | 20 < 19  (0.004) |
| Start Cath-Act | A < B  (0.011) |  |  |  | A < B  (0.014) |  |  |  | 20 < 19  (< 0.001) |
| Values are the p value of the one-sided test, and only significant results (< 0.05) are shown.  All abbreviations as in Table 1. | | | | | | | | | |

**S2.2 Prediction results**

A total of 1005 STEMI patients undergoing emergency PCI from 2018 to 2020 were selected for prediction, in which 155 with emergency bypass and 850 with non-emergency bypass. The time prediction mainly helps to optimize the existing treatment process of patients because the prediction is based on data of time intervals of previous STEMI patients satisfying the standard specifications (D-to-W ≤ 120 min for instance). Despite of presence of the unified specifications for the treatment process of STEMI patients, various hospitals may differ in the time taken in each stage. Therefore, different hospitals are able to adjust the treatment process before (when) treatment according to the predicted possible time consumption in the next stage, to rescue patients more reasonably and effectively.

**S3. More details of the Discussion**

**The analysis of D-to-W time**

STEMI is the most serious type of acute myocardial infarction (AMI) and its mortality remains very high although modern medical techniques have been used to race against time to rescue patients ^[2-4]^. Time of reperfusion therapy is critical for STEMI patients because a short ischemia time is associated with favorable clinical outcome and low acute and long-term mortality ^[5-8]^. Amid the ongoing COVID-19 pandemic, the treatment and management of STEMI patients have faced a dilemma because a compromise needs to be made between the timely reperfusion therapy and the rigorous prevention and control of COVID-19 infections ^[9,10]^.

1) In the scenario of a transfer–non-emergency bypass, the D-to-W_3_ time among interval variables in Group A is significantly longer than that in Groups B and C (p = 0.004, p = 0.004). This is probably because, in the initial outbreak of the pandemic, the coping strategies for the pandemic and scientific norms and procedures for sifting COVID-19 infections had not been made yet. In such context, COVID-19 infected patients could only be identified by using many complex methods and indicators including clinical symptoms, epidemiological survey, nucleic acid test results, neutrophil and lymphocyte counts in blood routine examination, and CT scans of the lungs. This substantially prolongs the time in the stage. After entering the normalized prevention and control period, canonical and concise procedures and contingency plans have been crafted. Especially, the application of the InterNet^+^ and big data analysis to the pandemic have significantly reduced the time needed for prevention and control of the COVID-19 pandemic.

2) In the scenario of non-emergency bypass, the interval variable D-to-W_5_ time in Groups A and B is obviously longer than that in Group C (p = 0.003, p < 0.001). After outbreak of the pandemic, the procedures and regulations related to pandemic prevention and control have been added to the stage compared with the pre-pandemic period. For example, epidemiological survey, relevant examination and inspection results, specialist consultation in pulmonology and infectious disease departments, and even nucleic acid test results need to be added in the stage. Emergency procedures and disinfection for pandemic prevention and control are introduced in the catheterization room. For patients suspected to be infected, a negative-pressure catheterization room even needs to be prepared; surgeons, assistants, and nurses in the catheterization room should use standard protective equipment and follow standard procedures. All of these increases the time of the subsection variable. After outbreak of the pandemic and in the prevention and control period of the pandemic, the pandemic prevention and control procedures are added in each time interval of the D-to-W time, so the total D-to-W time increases. This leads to the rise in-hospital mortality and incidence of heart failure ^[11-13]^. Therefore, new methods and procedures need to be innovated in a bid to reduce the delay in D-to-W amid the pandemic.

3) In the scenario of emergency bypass, results show that the interval variable FMC-to-Punc time in the Group 2020 during the COVID-19 pandemic is markedly shorter than the pre-pandemic Group 2019 (p = 0.006). This is because due to prevention and control measures during the COVID-19 pandemic, many STEMI patients choose to have a telemedicine-type interaction over the InterNet^+^ (WeChat groups of chest pain centers) in the diagnosis process. For example, the first-contact doctors in basic-level hospitals send clinical information and key examination and inspection results of patients to the InterNet^+^ before, and during the transfer process to interact with cardiovascular specialists in upper-level hospitals. The clinical information includes time of chest tightness or chest pain, consciousness and mental states, blood pressure, heart rate, breath state, and oxyhemoglobin saturation; the key examination and inspection results include blood routine examination, ECG, myocardial enzyme, and troponin. In this way, the authoritative diagnoses and treatment of upper-level hospitals move forward to this time interval in the transfer process, which greatly reduces the FMC-to-Punc time in the case of emergency bypass and improves the treatment efficiency of patients. This generates effects similar to those seen in other studies at present ^[14-16]^. Emergency bypass data show that even in the prevention and control period of the pandemic, the D-to-W time can also be reduced as long as more patients use the telemedicine interaction function of the InterNet^+^.

4) Favorable prediction results are attained for the scenario of emergency bypass, with the SMAPE basically below 0.6; the prediction results for the scenario of non-emergency bypass are also relatively good, with the coverage exceeding 0.75. In the scenario of a non-emergency bypass, the D-to-W_4_ time is poorly predicted, which is the only variable with the prediction indicator SMAPE exceeding 100%. This is probably because the time for deciding whether or not to intervene in cases involving Chinese patients is closely related to the judgement and decision of family members. However, family members exhibit greater individual differences than patients, which is related to many complex factors such as the family composition, level of education, experience, income, personal cultivation, and trust in hospitals and doctors, so the prediction results are poor. The InterNet^+^ has been applied to all patients undergoing emergency bypass, so the clinical manifestation, ECG, myocardial enzyme, and blood biochemical results of STEMI patients are all presented as pictures or texts to specialist physicians in upper-level hospitals in advance. As a result, the diagnosis of patients moves temporally and spatially forward, and each time node is within the controllable range. In addition, because the preoperative conversation with family members also moves forward to basic-level hospitals and ambulances, the stage with the longest delay (D-to-W_4_) is also significantly shortened in the case of an emergency bypass (p = 0.004).

**The Beneficial impact of InterNet+ in coping strategies**

The core to treatment of AMI is to minimize the time from disease onset to effective revascularization. With the standardized operation of chest pain centers, the in-hospital D-to-W time of patients has been substantially shortened and the mortality also declines correspondingly. For example, newly published research in 2022 acquired the precise living conditions of patients in chest pain centers by combining mortality surveillance data in China and data from chest pain centers. The in-hospital mortality and out-of-hospital mortality of 36,689 AMI patients admitted in 253 chest pain centers in 23 provincial-level administrative regions in China from 2019 to 2020 were studied. Previous research shows that the total in-hospital mortality of AMI patients was 4.0%, and that in second-level hospitals was 4.1%, which was significantly higher than 3.9% in tertiary hospitals. The total out-of-hospital mortality was 6.0%, and the out-of-hospital mortality in the second-level hospitals (7.8%) was higher than that in tertiary hospitals (5.2%). In comparison, multi-center registry studies in China in 2011 showed that the in-hospital mortality of AMI patients was as high as 10% at that time ^[17]^.

The standardized operation of each chest pain center before the COVID-19 pandemic has allowed a decrease in the mortality of AMI patients. Despite this, the out-of-hospital mortality is significantly higher than in-hospital mortality of AMI patients. In particular, the COVID-10 pandemic unavoidably prolongs the rescue time including the D-to-W time of AMI patients. In such context, strengthening out-of-hospital links of chest pain centers, paying attention to seamless pre-admission and in-hospital connection, and advancing the whole-process management system have become pressing issues. At present, the time from disease onset to FMC outside [primary](javascript:;) [hospital](javascript:;)s (basic-level hospitals) and the time from transfer to FMC in tertiary hospitals (upper-level hospitals) are significantly wasted and have large room to improve. This is attributed to the scarcity of medical resources in primary hospitals and low diagnosis and treatment levels and emergency consciousness therein. Although health and medical departments have made many efforts and implemented reforms, the situation will not be improved in the short-term. The further reduction of the in-hospital and out-of-hospital mortality of AMI patients and shortening of the time from disease onset to effective revascularization seem to hit a bottleneck, which is particularly prominent in the COVID-19 pandemic.

The National Chest Pain Center has introduced some special coping strategies and contingency plans in the pandemic prevention and control period. These, to some extent, relieved the huge impact of the pandemic on the treatment of AMI patients and reduced the otherwise prolonged D-to-W time amid the pandemic. Despite this, the D-to-W time is shortened in the scenario of emergency bypass while it is prolonged significantly in scenarios involving non-emergency bypass after the pandemic: this is mainly ascribed to the contribution of telemedicine interaction of the InterNet^+^. The research results indicate that the InterNet^+^ is a key to overcoming this bottleneck. Based on our questionnaire survey conducted among doctors at various levels of hospitals, it was found that there was an increased utilization of InterNet^+^ during the pandemic. Additionally, it was observed that patients with myocardial infarction received faster and more effective PCI treatment in hospitals, attributed to the shortened door-to-wire (D-to-W) time achieved through the application of InterNet^+^ tools (p = 0.019). This finding agrees with results of other observational studies and meta-analysis, which show that telemedicine can shorten the pre-hospital delays of patients receiving direct PCI ^[18-21]^. The telemedicine also provide additional clinical information, which can assist cardiologists in diagnosis and differential diagnosis with no need for an in-person diagnostic interview ^[22,23]^. However, the InterNet^+^ mentioned in the research is different from those specially developed telemedicine systems. In comparison, other specially developed telemedicine software has a high threshold to use and is expensive, making it inaccessible to primary hospitals and remote regions in developing countries. Based on public and free WeChat groups, some researchers investigated 24-h tele-ECG services, aiming to shorten the diagnosis and treatment time of STEMI patients. Passed from the pre-hospital tele-ECG service group in the WeChat groups, the results enable STEMI patients transferred from the PCI center to receive reperfusion earlier ^[24]^.

Key functions of the InterNet^+^ in the STEMI emergency treatment include: 1) Information arrives at specialists before patients. Transmission of patients’ information (ECG, symptoms, signs, and biochemical and examination results) allows rapid diagnosis and differential diagnosis of specialists in upper-level hospitals through interaction in the WeChat groups, to provide guidance in advance and collaborate in the treatment. 2) Doctors are prepared before the arrival of patients. The pre-hospital emergency is effectively linked to chest pain centers via the InterNet^+^. Then, medical resources are mobilized in the preparation process and are activated in advance (preparation of the catheterization room, preparation of members of operation groups, and pandemic prevention, control, and disinfection). 3) Mobile telemedicine guidance is provided in the emergency transport process. Guidance is provided for monitoring of ECG, blood pressure, and heart rate of patients in the ambulances during transport to realize seamless connection between pre-hospital emergency and fast channels in hospitals. 4) Preoperative conversations are brought forward; primary physicians and emergency physicians are guided to have conversation about the informed consent with patients and their family members in advance via interaction in the WeChat groups, which brings preoperative conversation temporally and spatially forward. This is a key point causing time delay in the operation of various chest pain centers at present.

The operation of the InterNet^+^ involves health care workers in relevant departments of all levels of hospitals and emergency physicians. When joining the WeChat groups, they have signed the confidentiality agreement for patient privacy. Specialist physicians in chest pain centers of upper-level hospitals take shifts around the clock to interact in the WeChat groups, which building an “expressway” for diagnoses and treatment of AMI patients. Interaction in the WeChat groups allows free use of texts, audio clips, videos, pictures, and phone calls, on the WeChat platform.

In summary, the InterNet^+^ as a simplified and free-charge telemedicine system, has the following two main functions: 1) It is key to reducing the effective rescue time of STEMI patients and therefore is worthy of further popularization, standardization, and improvement; 2) It also serves an effective tool for overcoming the bottleneck of time delay in STEMI rescue under the current COVID-19 pandemic prevention and control measures. Use of the InterNet^+^ by more patients should be advocated against the background of current normalized prevention and control measures for the pandemic. If the high-risk group of myocardial infarction can be managed by incorporating them in the telemedicine of the InterNet^+^ in the future, the effective control of the rescue time for myocardial infarction can be brought forward to the time from disease onset to FMC. This will open the last mile of the “expressway” for rescuing myocardial infarction patients and revolutionarily improving the prognosis of myocardial infarction.

**S4 Questionnaire questions and options**

**Survey of Internet+ use in STEMI**

Internet+ tool refers to the instant communication means between higher and lower hospitals, which can carry out telemedicine interaction in a timely and effective manner. Taking Wechat as an example, the Wechat communication group established based on the convenience and connectivity of the Internet can contact medical experts in superior hospitals with medical workers at lower levels and grassroots hospitals and even some cardiovascular patients with high-risk factors, which can effectively save the time for high-risk patients to go to primary hospitals and transfer to upper-level hospitals.

1. Is it a regional medical center (superior hospital) or grass-roots hospital (lower hospital)?

○ Regional Medical Center (Superior Hospital)

○ Primary Hospitals (lower-level hospitals)

2. What department are you working in now?

○ Cardiovascular Medicine -

○ Respiratory Medicine -

○ Emergency Medicine

○ Thoracic Surgery

○ Cardiac vascular surgery

○ Pulmonary Medicine

○ General Internal Medicine -

○ Other _________________

3. What is your title or position?

○ Senior Physician

○ Deputy Chief Physician

○ Attending Physicians

○ Treating physician

○ Deputy chief technician

○ Technician

○ Chief nurse

○ Deputy chief nurse

○ Supervisor nurse

○ Nurse Nurse

○ Nurse

○ Other _________________

4. What is your gender?

○ Male

○ Female

5. What's your age, please? [Fill in the blank] *

_________________________________

7. How often did you use Internet + before the epidemic?

○ Less every week

○ About 14 (2*7) times a week

○ About 21 (3*7) times a week

○ About 28 (4*7) times a week

○ About 35 (5*7) times a week

○ About 42 (6*7) times a week

○ More every week _________________

8. How often have you used Internet + since the outbreak?

○ Less every week

○ About 14 (2*7) times a week

○ About 21 (3*7) times a week

○ About 28 (4*7) times a week

○ About 35 (5*7) times a week

○ About 42 (6*7) times a week

○ More every week _________________

9. Do you think that the use of Internet+ can promote the optimization of diagnosis and treatment of STEMI?

○ Promoting effect

○ No promotion effect

○ Not sure

Please rate the contribution of Internet+ to the diagnosis and treatment optimization of STEMI [single choice] *

○ Very not important. ○ 20-30-40-50-60-80-90 - very important

Depends on the first choice of question 9.

What aspects do you think Internet+ can promote STEMI diagnosis and treatment? [multiple choice] *

□1. Provide myocardial infarction education and first aid information

□2. Provide telemedicine services

□3. Ecg monitoring and tracking

□4. Start the quick rescue network

□5. Other _________________

Depends on the first choice of question 9.

**References**

1. Rocha CN, Rodrigues F. Forecasting emergency department admissions. Intelligent Data Analysis, 2021 Jun;25(6):1579-1601.

2. Elbadawi A, Elgendy IY, Mahmoud K, et al. Temporal Trends and Outcomes of Mechanical Complications in Patients With Acute Myocardial Infarction. JACC Cardiovasc Interv. 2019 Sep 23;12(18):1825-1836. doi: 10.1016/j.jcin.2019.04.039.

3. Alabas OA, Jernberg T, Pujades-Rodriguez M, et al. Statistics on mortality following acute myocardial infarction in 842 897 Europeans. Cardiovasc Res. 2020 Jan 1;116(1):149-157. doi: 10.1093/cvr/cvz197.

4. Granger CB, Bates ER, Jollis JG, et al. Improving Care of STEMI in the United States 2008 to 2012. J Am Heart Assoc. 2019 Jan 8;8(1):e008096. doi: 10.1161/JAHA.118.008096.

5. Ibanez B, James S, Agewall S, et al. 2017 ESC Guidelines for the management of acute myocardial infarction in patients presenting with ST-segment elevation: The Task Force for the management of acute myocardial infarction in patients presenting with ST-segment elevation of the European Society of Cardiology (ESC). Eur Heart J. 2018 Jan 7;39(2):119-177. doi: 10.1093/eurheartj/ehx393.

6. Levine GN, Bates ER, Blankenship JC, et al. 2015 ACC/AHA/SCAI focused update on primary percutaneous coronary intervention for patients with ST-elevation myocardial Infarction: An update of the 2011 ACCF/AHA/SCAI guideline for percutaneous coronary intervention and the 2013 ACCF/AHA guideline for the management of ST-elevation myocardial infarction: A report of the American College of Cardiology/American Heart Association Task Force on Clinical Practice Guidelines and the Society for Cardiovascular Angiography and Interventions. Catheter Cardiovasc Interv. 2016 May;87(6):1001-19. doi: 10.1002/ccd.26325.

7. Wong GC, Welsford M, Ainsworth C, et al. 2019 Canadian Cardiovascular Society/Canadian Association of Interventional Cardiology Guidelines on the Acute Management of ST-Elevation Myocardial Infarction: Focused Update on Regionalization and Reperfusion. Can J Cardiol. 2019 Feb;35(2):107-132. doi: 10.1016/j.cjca.2018.11.031.

8. Foo CY, Bonsu KO, Nallamothu BK, et al. Coronary intervention door-to-balloon time and outcomes in ST-elevation myocardial infarction: a meta-analysis. Heart. 2018 Aug;104(16):1362-1369. doi: 10.1136/heartjnl-2017-312517.

9. Scholz KH, Maier SKG, Maier LS, et al. Impact of treatment delay on mortality in ST-segment elevation myocardial infarction (STEMI) patients presenting with and without haemodynamic instability: results from the German prospective, multicentre FITT-STEMI trial. Eur Heart J. 2018 Apr 1;39(13):1065-1074. doi: 10.1093/eurheartj/ehy004.

10. Zeng J, Huang J, Pan L. How to balance acute myocardial infarction and COVID-19: the protocols from Sichuan Provincial People's Hospital. Intensive Care Med. 2020 Jun;46(6):1111-1113. doi: 10.1007/s00134-020-05993-9.

11. Xiang D, Xiang X, Zhang W, et al. Management and Outcomes of Patients With STEMI During the COVID-19 Pandemic in China. J Am Coll Cardiol. 2020 Sep 15;76(11):1318-1324. doi: 10.1016/j.jacc.2020.06.039.

12. De Luca G, Verdoia M, Cercek M, et al. Impact of COVID-19 Pandemic on Mechanical Reperfusion for Patients With STEMI. J Am Coll Cardiol. 2020 Nov 17;76(20):2321-2330. doi: 10.1016/j.jacc.2020.09.546.

13. Chew NWS, Ow ZGW, Teo VXY, et al. The Global Effect of the COVID-19 Pandemic on STEMI Care: A Systematic Review and Meta-analysis. Can J Cardiol. 2021 Sep;37(9):1450-1459. doi: 10.1016/j.cjca.2021.04.003.

14. Nan J, Jia R, Meng S, Jin Y, Chen W, Hu H. The Impact of the COVID-19 Pandemic and the Importance of Telemedicine in Managing Acute ST Segment Elevation Myocardial Infarction Patients: Preliminary Experience and Literature Review. J Med Syst. 2021 Jan 3;45(1):9. doi: 10.1007/s10916-020-01703-6.

15. Krishnamoorthy P, Vengrenyuk A, Wasielewski B, et al. Mobile application to optimize care for ST-segment elevation myocardial infarction patients in a large healthcare system, STEMIcathAID: rationale and design. European Heart Journal-Digital Health. 2021 Feb;2(2):189–201.

16. Zhang AAY, Chew NWS, Ng CH, et al. Post-ST-Segment Elevation Myocardial Infarction Follow-Up Care During the COVID-19 Pandemic and the Possible Benefit of Telemedicine: An Observational Study. Front Cardiovasc Med. 2021 Oct 22;8:755822. doi: 10.3389/fcvm.2021.755822.

17. Nan J, Meng S, Hu H, et al. Comparison of Clinical Outcomes in Patients with ST Elevation Myocardial Infarction with Percutaneous Coronary Intervention and the Use of a Telemedicine App Before and After the COVID-19 Pandemic at a Center in Beijing, China, from August 2019 to March 2020. Med Sci Monit. 2020;26:e927061. Published 2020 Sep 17. doi:10.12659/MSM.927061

18. Long Z, Liu W, Zhao Z, et al. Case Fatality Rate of Patients with Acute Myocardial Infarction in 253 Chest Pain Centers - China, 2019-2020. China CDC Wkly. 2022 Jun 17;4(24):518-521. doi: 10.46234/ccdcw2022.026.

19. Brunetti ND, Dell'Anno A, Martone A, et al. Prehospital ECG transmission results in shorter door-to-wire time for STEMI patients in a remote mountainous region. Am J Emerg Med. 2020 Feb;38(2):252-257. doi: 10.1016/j.ajem.2019.04.046.

20. Goebel M, Bledsoe J. Push Notifications Reduce Emergency Department Response Times to Prehospital ST-segment Elevation Myocardial Infarction. West J Emerg Med. 2019 Mar;20(2):212-218. doi: 10.5811/westjem.2018.12.40375.

21. Brunetti ND, De Gennaro L, Correale M, et al. Pre-hospital electrocardiogram triage with telemedicine near halves time to treatment in STEMI: A meta-analysis and meta-regression analysis of non-randomized studies. Int J Cardiol. 2017 Apr 1;232:5-11. doi: 10.1016/j.ijcard.2017.01.055.

22. Baker J, Stanley A. Telemedicine Technology: a Review of Services, Equipment, and Other Aspects. Curr Allergy Asthma Rep. 2018 Sep 26;18(11):60. doi: 10.1007/s11882-018-0814-6.

23. Weinstein RS, Krupinski EA, Doarn CR. Clinical Examination Component of Telemedicine, Telehealth, mHealth, and Connected Health Medical Practices. Med Clin North Am. 2018 May;102(3):533-544. doi: 10.1016/j.mcna.2018.01.002.

24. Liu H, Wang W, Chen H, Li Z, Feng S, Yuan Y. Can WeChat group-based intervention reduce reperfusion time in patients with ST-segment myocardial infarction? A controlled before and after study. J Telemed Telecare. 2020 Dec;26(10):627-637. doi: 10.1177/1357633X19856473.
